# Supplementary figures and images for: Comparative Transcriptomic Analysis of Cerebellar Astrocytes across Developmental Stages and Brain Regions
Source: Int J Mol Sci. 2024 Jan 13;25(2):1021. doi: 10.3390/ijms25021021 (PMC10816327; doi:10.3390/ijms25021021)

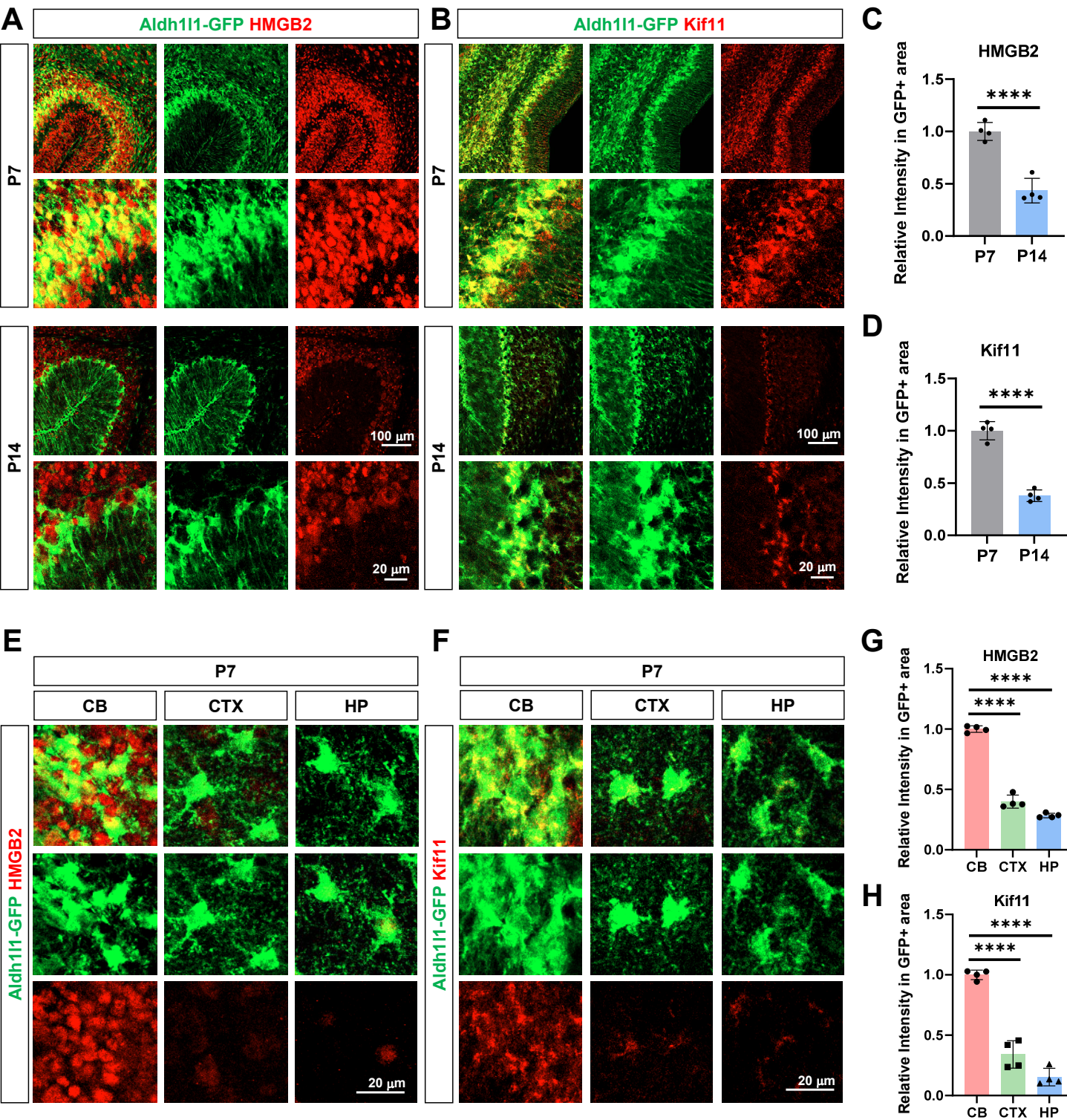

Supplement: Supplementary file 1 [file ijms-25-01021-s001.zip › Supplementary Figure 1.pdf]
